# Peer review analysis in the field of radiation oncology: results from a web-based survey of the Young DEGRO working group

**DOI:** 10.1007/s00066-020-01729-2

**Published:** 2020-12-18

**Authors:** Lukas Käsmann, Annemarie Schröder, Benjamin Frey, Daniel F. Fleischmann, Tobias Gauer, Nadja Ebert, Markus Hecht, David Krug, Maximilian Niyazi, Matthias Mäurer, Young DEGRO Group

**Affiliations:** 1 Department of Radiation Oncology, LMU University Hospital, Marchioninistraße 15, 81377 Munich, Germany; 2 Comprehensive Pneumology Center Munich (CPC-M), Member of the German Center for Lung Research (DZL), Munich, Germany; 3 German Cancer Consortium (DKTK), partner site Munich, Munich, Germany; 4 Department of Radiation Oncology, University Hospital Rostock, Rostock, Germany; 5 Department of Radiation Oncology, Universitätsklinikum Erlangen, Erlangen, Germany; 6 German Cancer Research Center (DKFZ), Heidelberg, Germany; 7 Department of Radiation Oncology, University Hospital Hamburg-Eppendorf, Hamburg, Germany; 8 OncoRay-National Center for Radiation Research in Oncology, Medical Faculty and University Hospital Carl Gustav Carus, Technische Universität Dresden, Dresden, Germany; 9 Department of Radiation Oncology, University Hospital Schleswig-Holstein campus Kiel, Kiel, Germany; 10 Department of Radiation Oncology, University Hospital Jena, Jena, Germany

**Keywords:** Radiation oncology, Peer review, Publication, Impact factor, Scientific publishing

## Abstract

**Purpose:**

To evaluate the reviewing behaviour in the German-speaking countries in order to provide recommendations to increase the attractiveness of reviewing activity in the field of radiation oncology.

**Methods:**

In November 2019, a survey was conducted by the Young DEGRO working group (jDEGRO) using the online platform “eSurveyCreator”. The questionnaire consisted of 29 items examining a broad range of factors that influence reviewing motivation and performance.

**Results:**

A total of 281 responses were received. Of these, 154 (55%) were completed and included in the evaluation. The most important factors for journal selection criteria and peer review performance in the field of radiation oncology are the scientific background of the manuscript (85%), reputation of the journal (59%) and a high impact factor (IF; 40%). Reasons for declining an invitation to review include the scientific background of the article (60%), assumed effort (55%) and a low IF (27%). A double-blind review process is preferred by 70% of respondents to a single-blind (16%) or an open review process (14%). If compensation was offered, 59% of participants would review articles more often. Only 12% of the participants have received compensation for their reviewing activities so far. As compensation for the effort of reviewing, 55% of the respondents would prefer free access to the journal’s articles, 45% a discount for their own manuscripts, 40% reduced congress fees and 39% compensation for expenses.

**Conclusion:**

The scientific content of the manuscript, reputation of the journal and a high IF determine the attractiveness for peer reviewing in the field of radiation oncology. The majority of participants prefer a double-blind peer review process and would conduct more reviews if compensation was available. Free access to journal articles, discounts for publication costs or congress fees, or an expense allowance were identified to increase attractiveness of the review process.

**Supplementary Information:**

The online version of this article (10.1007/s00066-020-01729-2) contains supplementary material, which is available to authorized users.

## Introduction

The field of radiation oncology is continuously changing due to development of new radiation delivery and planning techniques, new systemic anticancer agents and demographic changes. Scientific journals have the important goal of disseminating these latest study results and contribute to the cumulative knowledge of the scientific community. With an increasing number of oncology journals listed in the Journal Citation Reports [1, 2], from 143 in 2008 to 230 in 2018, continuous and high-quality reviewing assessment is becoming more and more important. The increasing importance of the impact factor (IF) needs to be considered for quality and the number of manuscript submissions as well as for the motivation to contribute as a reviewer [3, 4]. At the same time, smaller or regional journals with a lower IF may encounter more difficulties in maintaining a proper review process. Based on the analysis of 3500 review experiences, Huisman et al. revealed that the satisfaction of authors as well as reviewers depends on the journal’s IF and duration of the review process [5]. Importantly, higher-ranked journals seemed to be more effective concerning the duration of the first review round, total review duration and immediate rejection time [5].

In the present survey, we aimed to evaluate and analyse the reviewing behaviour in German-speaking countries (Austria, Switzerland and Germany) in the field of radiation oncology. Based on the analysis, we sought to identify current trends and challenges in order to develop recommendations to increase the attractiveness of peer review activity.

## Methods

A web-based questionnaire was developed within the survey platform “eSurveyCreator.com” (enuvo GmbH, Seefeldstraße 25, 8008 Zurich, Switzerland) containing 29 items regarding demographics and experience in publishing and reviewing scientific articles in the field of radiation oncology (see Supplementary I). Branching logic was used to tailor the questions on the basis of previous responses.

The data sample was collected through an online anonymized survey of radiation oncologists, biologists and physicists in German-speaking countries (Austria, Switzerland and Germany). In order to broadly address eligible participants, invitations were initially sent to members of the German Society of Radiation Oncology (DEGRO), German Society for Biological Radiation Research (DeGBS), German Society for Medical Physics (DGMP), Austrian Society of Radiooncology (ÖGRO) and the Swiss Society of Radiation Oncology (SRO) as well as through direct peer-to-peer contacts. A first invitation was sent to all participants on 8 October 2019 and a reminder email on 29 October 2019 to maximize the response rate. The survey was closed for any contribution on 24 November 2019. Multiple responses to the survey were prevented by locking the participants IP (internet protocol) address and setting cookies.

Raw data of the results were received directly from the online platform and were analysed using Microsoft Excel (Microsoft version 2001, Microsoft, Redmond, WA, USA) and SPSS (Mac version 25, IBM SPSS Statistics, IBM, Armonk, NY, USA).

## Results

We received a total of 281 responses from 8 October to 24 November 2019. Of these, 154 (55%) questionnaires were completed and included in the evaluation.

### General aspects and demographics

Table 1 summarizes participants’ characteristics and demographics. The median age group of the participants was 41–50 years. 37% of respondents were female and 63% male. 51% of the participants were physicians, 16% biologists, 27% medical physicists and 6% work in other fields. Of these, 83% work in Germany, 10% in Switzerland and 5% in Austria. The majority of participants were postdoctoral researchers (45%), habilitated faculty members (21%) or appointed professors (24%). The majority of participants (53%) have 5–20 years of experience in publishing scientific articles (Table 2). Every participant has published a mean value of 55 (95% confidence interval, CI, 41–69) scientific articles. In 2018, participants published a median of one article (range 0–24) as a first author, three articles (range 0–30) with co-authorship and one article (range 0–15) as the last author.

**Table 1:** General data and demographics

Parameter	*N* (%)
*Gender*	
Female	57 (37)
Male	97 (63)
*Age*	
20–25 years	1 (1)
26–30 years	12 (8)
31–35 years	24 (16)
36–40 years	21 (14)
41–50 years	39 (25)
51–60 years	41 (27)
61–70 years	12 (8)
>70 years	4 (3)
*Professional background*	
Medicine	79 (51)
Physics	42 (27)
Biology	24 (16)
Others	9 (6)
*Academic level*	
MD student	6 (4)
PhD student	9 (6)
Postdoctoral researcher	69 (45)
Habilitation	33 (21)
Professorship	37 (24)
*Country*	
Germany	127 (83)
Switzerland	15 (10)
Austria	7 (5)
Others	5 (2)

*MD* medical, *PhD* Doctor of Philosophy

**Table 2:** Overview of publishing and reviewing characteristics

Parameter	*N* (%)
*Experience in scientific publishing*	
<5 years	23 (15)
5–10 years	27 (18)
11–15 years	26 (17)
16–20 years	28 (18)
21–30 years	21 (14)
>30 years	14 (9)
Unknown	12 (8)
*Preferred peer review method*	
Double-blind	106 (70)
Single-blind	24 (16)
Open review	21 (14)
No opinion	3 (1)
*Number of reviews in 2018*	
0	33 (21)
1–4	45 (29)
5–12	53 (34)
13–14	15 (10)
>24	4 (3)
Unknown	4 (3)
*Rewarded for writing a peer review*	
Yes	18 (12)
No	127 (82)
Unknown	9 (6)

The most common platform to showcase scientific experience and publications was Researchgate (80%). Other platforms used by the participants are ORCID (58%), Scopus (23%) and Publons (16%).

### Peer review

**Fig. 1:**
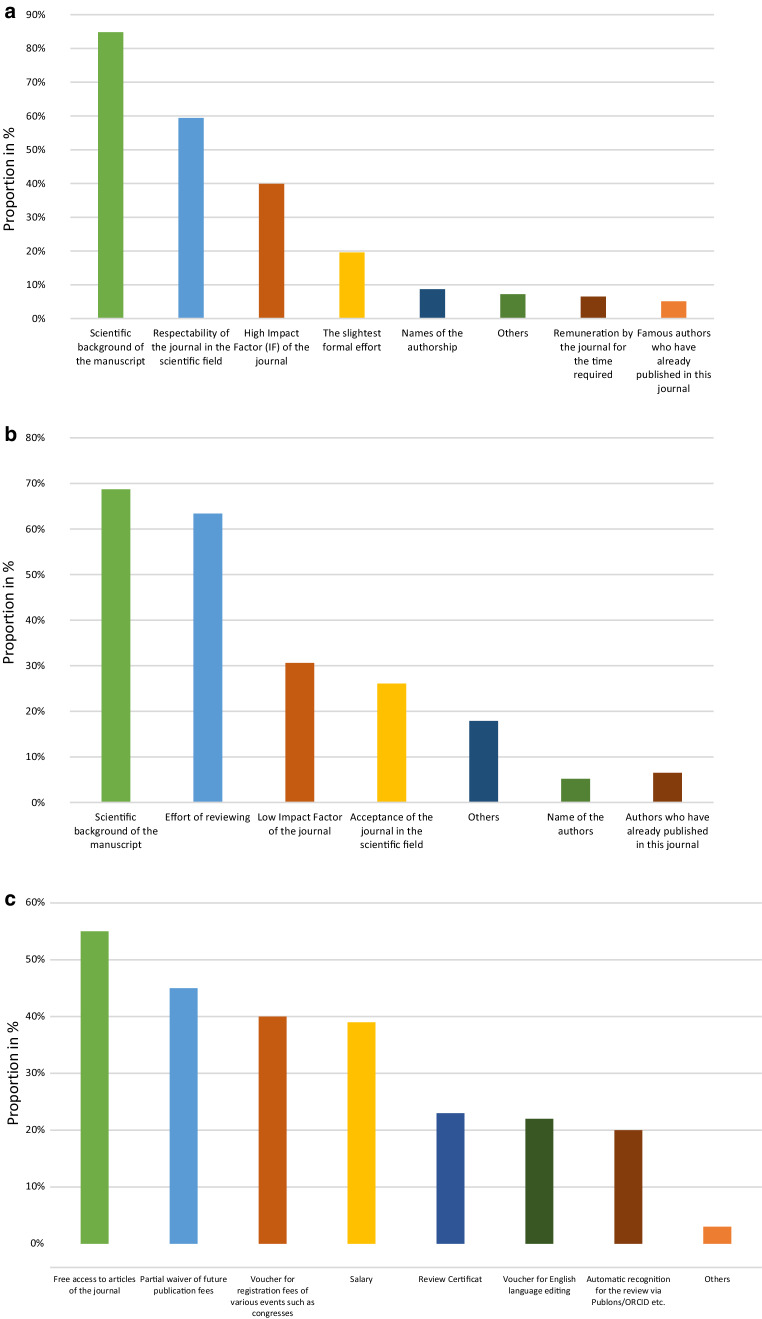
**a** Important criteria for performing peer review by participants (*n* = 138). **b** Important criteria for declining peer review by participants (*n* = 138). **c** Compensations/rewards preferred by the participants for writing a peer review (*n* = 138)

**Fig. 2:**
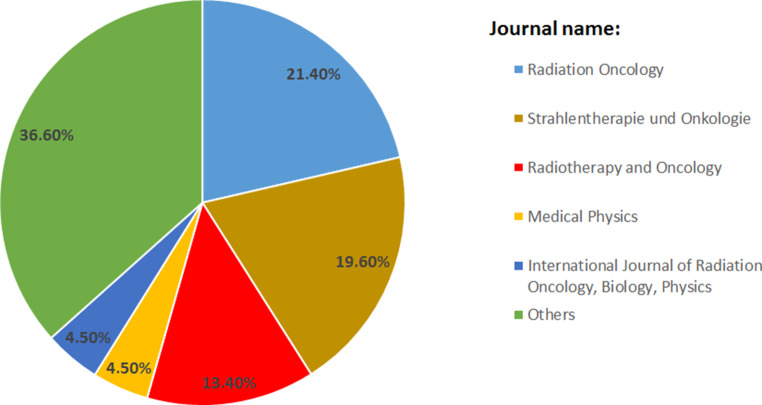
Selected journals which are most reviewed by participants. Others: Cumulative percentage of all journals mentioned by less than 3% of all participants

The most important factors for journal selection and peer review performance in the field of radiation oncology are the scientific background of the manuscript (85%), reputation of the journal (59%) and a high IF (40%; Fig. 1a). Reviewers older than 35 years and those having more than 10 years’ experience in scientific publishing in radiation oncology reviewed more manuscripts in 2018 (86 vs. 54, *p* < 0.001; 90 vs. 56, *p* < 0.001) than younger reviewers and reviewers with less experience. In 2018, the majority (63%) reviewed 1 to 12 manuscripts. Profession was not associated with reviewing (*p* = 0.634). Furthermore, male participants performed more peer reviews compared to female participants (33% of all female and 14% of all male participants performed no reviews in 2018, *p* = 0.005). 54.4% of all participants indicated that *Radiation Oncology, Strahlentherapie und Onkologie* or *Radiotherapy and Oncology* are the most common journals where participants perform reviewing (Fig. 2). 21% of all participants reviewed no submitted articles in 2018, of whom 64% would perform peer review if compensations were offered.

Each participant declined on average 11 (95% CI: 6–15) invitations to review a manuscript in 2018. The most important criteria for declining an invitation were the scientific background of the manuscript (60%), effort of reviewing (e.g., needed time, missing compensation; 55%) and low IF of the journal (27%; Fig. 1b).

Overall, 70% of the participants prefer a double-blind review process over a single-blind (16%) or an open review process (14%; see Table 2). Peer review and editorial contributions to academic journals are shown mainly on Researchgate (31%) and ORCID (24%). 12% of the participants have been rewarded for writing a peer review. This included partial waiver of future publication fees (67%), free access to articles of the journal (56%), salaries (6%), voucher for registration fees of various events such as scientific meetings (6%) and other (22%). 59% of all participants would review more frequently if some kind of compensation/reward was offered. Experience in scientific publishing is associated with willingness to perform peer review (*p* = 0.006). Participants with less than 15 years of experience in scientific publishing would review more frequently if receiving rewards when compared to participants with more experience (*p* = 0.025). Compensations preferred by the participants for writing a peer review are free access to journal articles (55%), partial waiver of future publication fees (45%), voucher for registration fees of various events such as scientific meetings (40%), salary (39%), review certificate (23%), voucher for English language editing (22%), automatic recognition for the review via Publons/ORCID etc. (20%) and others (3%; Fig. 1c).

## Discussion

The vast majority of researchers consider peer review an integral part of their work and the scientific communication system [6, 7]. The peer review of manuscripts is often considered an altruistic duty of members of the research community [8]. However, the current scientific peer review process faces several obstacles such as increasing number of journals, submissions and non-adequate referee reports. To address these challenges, we conducted the first survey evaluating peer review activity in German-speaking countries (Austria, Switzerland and Germany), in order to develop recommendations to increase the attractiveness of peer review in the field of radiation oncology.

Our survey demonstrates that the most important criteria for peer reviewing are the scientific background of the manuscript (85%), the reputation of the journal (59%) and a high IF (40%). These findings are consistent with recent literature underlining the important role of the IF and the respectability of the journal for reputation, funding and career development [9, 10]. However, the suitability of the manuscript for the reviewer seems to be the most important criterion and should be considered in order to improve peer review activity in radiation oncology.

The majority of the participants (70%) of our survey prefer a double-blind review process over single-blind or open review. These findings go along with the published literature [6, 11]. Interestingly, only 14% would prefer the new concept of an “open review”. In fact, “open review” is described by more than 120 definitions in literature [12]. As a result, several characteristics may be incorporated, such as an open interaction between readership, authors and reviewers, non-blinded reviewing, reports and participation to the manuscript [12]. Single- or double-blind reviews have been suggested to protect reviewers from the author’s influence, allowing them to give objective feedback and thus minimizing the potential for tortious interference. Several studies have failed to show that single- or double-blind reviewing increases the quality of the review process [13–15]. Due to effective online search methods and close scientific communities, blinded reviewing has been found to successfully conceal the author’s identity in only 54–74% of all cases [13, 14].

The increasing workload placed on peer reviewers due to a rapidly growing volume of publications and inappropriate selection of reviewers by the editorial board are frequently discussed [16]. Therefore, identifying qualified reviewers and keeping them motivated for future submissions is an important editorial task. Well-known researchers may be asked several times a day for their review contribution, resulting in a delay in the review process and decline of authors’ submissions due to increased processing time. An effective, timely review process is associated with higher IFs and satisfaction of authors and reviewers [5, 17]. In contrast to other studies, our survey revealed the important role of the scientific background of the manuscript (69%) and the effort of reviewing (63%) as the main reasons for declining peer review. With regard to the significance of the IF, our data is consistent with the results of the Global State of Peer Review Report 2018 [18].

The association between the likelihood of accepting an invitation for review and its scientific background as well as the choice of a journal based on high IF and visibility in the field creates particular strain for journals with a lower IF. Most manuscripts will at first be submitted to higher-IF journals where many of them will undergo a first round of peer review [19]. In case of rejection, they will then be re-submitted to journals with a lower IF, which—according to our analysis—will be less likely be able to facilitate a timely peer review process due to their lower attractiveness for potential peer reviewers and the increased workload due to several independent rounds of peer review.

High-IF journals perform a rigorous preliminary selection before external peer review, resulting in fewer review requests and a more effective peer review process. In order to improve the peer review process, pre-selection by the editorial office would result in significantly reduced review times. However, preliminary selection may undermine the neutral stance of the peer review process.

Especially for young researchers, the invitation to review a research article based on the recognition of expertise can be considered a great honour and important part on the way to becoming an independent researcher. However, as our results show, particularly established participants over 35 years of age with more than 10 years of experience in scientific publishing are willing to review manuscripts on a regular basis. A possible cause, according to younger scientists, could be the lack of relevance of peer reviewing for advancing their career and the lack of compensation for the often time-consuming work. Instead of conducting peer review, young scientists may be more interested in publishing their own research results and increasing their reputation. In addition to the reduced level of scientific expertise, this could explain why the participants were mainly postdoctoral researchers (45%), habilitated scientists (21%) and professors (24%). This hypothesis is underlined by survey results from Nicholson et al. [20], where about 70% of respondents indicated that they would include peer review as a professional service in their curriculum vitae. Importantly, 27% of respondents cited formal recognition in assessment as a factor that would motivate them to participate in public peer reviews. The results may also demonstrate a growing sense of frustration that for-profit companies, i.e., publishers, benefit in several ways from the scientific community. First, they receive direct or indirect payments for publication and for access to these articles. Second, peer review is conducted at no charge. In recent years, article-processing charges (APCs) have increased dramatically for several publishers, particularly open-access or hybrid open-access publishers, without any rewards for reviewers, resulting in enhancement of this questionable situation. As a result, predatory publishing has become a popular business model that involves charging publication fees to authors without providing editorial and publishing services or quality checks, which legitimate academic journals provide.

In our survey, only 12% of the participants have been rewarded for writing a peer review, namely partial waiver of publication fees (67%), free access to articles of the journal (56%), salary (6%) and vouchers for registration fees of various events such as scientific meetings (6%). Importantly, 59% of all participants stated that they would review more frequently if some kind of compensation or reward were offered. A Wiley study with more than 2900 participants revealed that reviewers are not satisfied regarding recognition for their work and stated that they should be rewarded for their effort [17].

Our survey demonstrates that scientific experience is correlated to review activity. However, due to the rising submission rates, we see an urgent need to actively engage early career researchers in the review process. In order to facilitate and promote peer review, peer review trainings for young researchers may improve both the motivation for and the quality of peer review reports [17]. The suitability of the reviewer for the submitted manuscript seems to be the most important criterion for peer review performance and should be critically recognized.

The results of this survey must be interpreted in light of its limitations: non-responders and participants with incomplete surveys (127 of 281 [45%]) might have answered differently and, therefore, our findings cannot be applied to a general statement. However, we believe that the diversity of our responder characteristics concerning age, gender, profession and scientific publishing expertise is comparable to the scientific community in radiation oncology and increases the informative value. Moreover, the used Likert scales may capture only broad perspectives in this complex issue of scientific publishing.

In summary, the suitability of the reviewer and the scientific background of the manuscript are the crucial criteria for willingness to peer review. Therefore, rigorous preliminary selection before external peer review may result in fewer review requests, more manuscripts that are suitable and a more effective peer review process. The review procedure should be performed in a double-blind manner. Rewards for peer review activity such as partial waiver of publication fees and free access to articles of the journal would increase the willingness to peer review. Peer review trainings for young researchers may improve both the motivation for and the quality of peer review reports.

## Conclusion

Peer review activity in radiation oncology is determined by the suitability of the reviewer, reputation and a high IF of the journal. Rigorous pre-selection may result in fewer review requests and more effective peer review process. A double-blind peer review process is recommended by the majority of the participants. Review activity would increase if compensation were available. Free access to journal articles, discounts for publication costs or congress fees, or an expense allowance are ways to make the review process more attractive.

## Supplementary Information

Supplementary I Survey questions/statements
