# Supplementary material for: Peer review analysis in the field of radiation oncology: results from a web-based survey of the Young DEGRO working group
Source: Strahlenther Onkol. 2020 Dec 18;197(8):667–73. doi: 10.1007/s00066-020-01729-2 (PMC8292256; doi:10.1007/s00066-020-01729-2)
Supplement: Supplementary file 1 — Supplementary I Survey questions/statements [file 66_2020_1729_MOESM1_ESM.pdf]

Reviewing and publication in the field of radiation oncology

Page 1

Dear colleagues,
in this short survey, we want to investigate the publication and reviewing behaviour in the field of radiation oncology. Obtaining feedback from authors and reviewers is vital to the review process and helps us improving and developing new innovative ideas and formats. Let your voice be heard. We would be very grateful if you could take the time to complete the following survey. It should take about five minutes of your time.
Your responses are voluntary and will be confidential. Responses will not be identified by individual. All responses will be compiled together and analyzed as a group.
If you have any questions or concerns, please contact us via mail jd@degro.org (subject: Survey)

Thank you very much,
in behalf of the jDEGRO (working group of the German Society of Radiation Oncology (DEGRO e.V.))
Dr. med. Lukas Käsmann

Page 2

What is your gender? *

- ◯ female
- ◯ male

What is your age? *

Please choose...

What is your professional background? *

- ◯ Medicine
- ◯ Biology
- ◯ Physics
- ◯ ____________

What is your academic level?

Please choose...

In which country do you work? *

Please choose...

Page 3

In which federal state do you work? *

Please choose...

Page 4

For how long have you been publishing in scientific journals?

Please choose...

Do you use platforms to showcase your publications, e.g. Researchgate?

- [ ] Researchgate: Social networking site for scientists and researchers to share papers, ask and answer questions, and find collaborators.
- [ ] Publons: Website to track, verify and showcase peer review activity and editorial contributions for academic journals
- [ ] Scopus: larges abstract and citation database of peer-reviewed literature: scientific journals, books and conference proceedings.
- [ ] ORCID: provides a persistent digital identifier that simplefy manuscript and grant submission, supports automated linkages about professional activities/publications.
- [ ] Loop (Frontiers Journal Series): social network for researchers, academics and scholars
- [ ] Google Scholar: large web search engine that indexes the full text or metadata of scholarly literature across an array of publishing formats and disciplines
- [ ] ______

How many manuscripts have you submitted in 2018...?

...as a first author (with or without shared first authorship) [ ]

...as a corresponding author? [ ]

...as a co-author? [ ]

...as a senior author? [ ]

Page 5

How many of your scientific contributions have been published in total so far?

[ ]

How do you choose a journal in which to submit a manuscript? What is the most important criterion for choosing a journal for submission?

1=most important, 10=least important

- Minimal technical / formal requirements
- The highest possible Impact Factor
- Famous authors who have already published in this journal
- Acceptance of the journal in the scientific field
- The shortest possible time from submission to decision
- Matching Special Issues of a certain journal
- Possibility to publish with open access
- Invitations from the Journal
- Based on lowest publication fee
- Visibility within your scientific community

Which kind of review procedure do you prefer?

double-blind review: The reviewer and author identities are concealed from the each other, and vice versa, throughout the review process.
single-blind peer review: The identity of the reviewer is anonymous, but the author's name and affiliation are on the paper.
Open peer review: The identity of the author and the reviewers are known by the author, reviewer and the readers.

- ◯ double-blind review
- ◯ single-blind peer review
- ◯ open peer review

How many manuscripts did you review in 2018?

Please choose...

Page 6

Have you served as a reviewer in one of these journals in 2017 or 2018?
In which journals have you served the most as reviewer?

Journals are listed in alphabetical order and provide medical journals (oncology/radiotherapy/radiology). In the next questions you will be asked about publication in biology and physics!

Please choose...

In which journals have you served the second most as reviewer?

Journals are listed in alphabetical order and provides medical journals (oncology/radiotherapy/radiology). In the next questions you will be asked about publication in biology and physics!

Please choose...

In which journals have you served the third most as reviewer?

Journals are listed in alphabetical order and provides medical journals (oncology/radiotherapy/radiology). In the next questions you will be asked about publication in biology and physics!

Please choose...

You could not find your medical journal in the list? If yes, please provide the medical journal in which you served as a reviewer in 2017 or 2018 in the free text field:

Please avoid any abbreviation!

the most

the second most

the third most

Did you serve as a reviewer in the field of biology? If yes, please provide the biology journals in which you served as a reviewer in 2017 or 2018 in the free text field:

Please avoid any abbreviation!

the most

the second most

the third most

Did you serve as a reviewer in the field of physics? If yes, please provide the physics journals in which you served as a reviewer in 2017 or 2018 in the freetext:

Please avoid any abbreviation!

the most

the second most

the third most

In which journals did you serve as a reviewer in 2017 or 2018 as well?

How often did you decline an invitation to review a manuscript in 2018?

[ ]

What are your criteria to decline the effort of reviewing a manuscript?

- [ ] Low Impact Factor of the journal
- [ ] Effort of reviewing (eg. needed time, missing compensation)
- [ ] scientific background of the manuscript
- [ ] Acceptance of the journal in the scientific field
- [ ] Authors who have already published in this journal
- [ ] Names of the authors
- [ ] ____

What are your criteria to accept the effort of reviewing a manuscript?

- [ ] high Impact Factor of the journal
- [ ] Respectability of the journal in the scientific field
- [ ] Famous authors who have already published in this journal
- [ ] The scientific background of the manuscript fits with your own scientific field
- [ ] The slightest formal effort (eg. Time sparing, short template)
- [ ] Remuneration by the magazine for the time required (eg. Discount for next submission fee)
- [ ] Names of the authorship
- [ ] ____

Which platforms do you use to showcase your peer review and editorial contributions for academic journals?

Researchgate: Social networking site for scientists and researchers to share papers, ask and answer questions, and find collaborators.
Publons: Website to track, verify and showcase peer review activity and editorial contributions for academic journals
Scopus: larges abstract and citation database of peer-reviewed literature: scientific journals, books and conference proceedings.
ORCID: provides a persistent digital identifier that simplefy manuscript and grant submission, supports automated linkages about professional activities/publications.
Loop (Frontiers): social network for researchers, academics and scholars
Google Scholar: large web search engine that indexes the full text or metadata of scholarly literature across an array of publishing formats and disciplines

	yes	no
Researchgate	◯	◯
Publons	◯	◯
Scopus	◯	◯
ORCID	◯	◯
Loop (Frontiers)	◯	◯
Google Scholar	◯	◯

Have you ever been rewarded for writing a peer review?

◯ yes

◯ no

Page 9

How were you rewarded by the Journal?

☐ Partial waiver of future publication fees

☐ Voucher for English language editing

☐ Voucher for registration fees of various events such as congresses

☐ Free access to articles of the journal

☐ Salary

☐ ____________

Page 10

Would you review more frequently if some kind of compensation/reward was offered?

- ○ yes
- ○ no

What kind of compensation/reward would you prefer?

- ☐ Partial waiver of future publication fees
- ☐ Voucher for English language editing
- ☐ Voucher for registration fees of various events such as congresses
- ☐ Free access to articles of the journal
- ☐ Automatic recognition for the review via Publons/ORCID etc.
- ☐ Salary
- ☐ Review Certificat
- ☐ ____________

What should be changed by scientific journals to improve the submission/review process?

Please feel free to comment (non-mandatory)!

» **Redirection to final page of SurveyHero (formerly eSurveyCreator)** (change)
